# Is Loneliness a Cause or Consequence of Dementia? A Public Health Analysis of the Literature

**DOI:** 10.3389/fpsyg.2020.612771

**Published:** 2021-02-18

**Authors:** Christina R. Victor

**Affiliations:** College of Health, Medicine and Life Sciences, Brunel University London, Uxbridge, United Kingdom

**Keywords:** loneliness, dementia, longitudinal studies, measurement of loneliness, measurement of dementia, public health

## Abstract

Loneliness has been reframed from a ‘social problem of old age’ into a major public health problem. This transformation has been generated by findings from observational studies of a relationship between loneliness and a range of negative health outcomes including dementia. From a public health perspective, key to evaluating the relationship between loneliness and dementia is examining how studies define and measure loneliness, the exposure variable, and dementia the outcome. If we are not consistently measuring these then building a body of evidence for the negative health outcomes of loneliness is problematic. Three key criteria had to meet for studies to be included in our analysis. To test the proposition that loneliness is a cause of dementia we only included longitudinal studies. For inclusion studies had to measure loneliness at baseline, have samples free of dementia and assess dementia at follow up (specified as a minimum of 12 months). We identified 11 papers published between 2000 and 2018 that meet these criteria. These studies included seven different countries and only one was specifically focused upon dementia: all other studies were cohort studies focused upon ageing and health and wellbeing. There was extensive heterogeneity in how studies measured loneliness and dementia and in the use of co-variates. Loneliness was measured by either self-rating scales (*n* = 8) or scales (*n* = 3). Dementia was assessed by clinical tests (*n* = 5), diagnostic/screening tools (*n* = 3), cognitive function tests (*n* = 1), and self-reported doctor diagnosis (*n* = 2). Substantial variation in loneliness prevalence (range 5–20%) and dementia incidence (5–30 per 1000 person years at risk). Six studies did not report a statistically significant relationship between loneliness and dementia. Significant excess risk of dementia among those who were lonely ranged from 15% to 64%. None of these studies are directly comparable as four different loneliness and dementia measures were used. We suggest that the evidence to support a relationship between loneliness and dementia is inconclusive largely because of methodological limitations of existing studies. If we wish to develop this evidence base, then using a consistent set of loneliness and dementia outcome measures in major longitudinal studies would be of benefit.

## Introduction

Loneliness is experienced across the lifespan (ONS, 2018) and across cultures (Barreto et al., 2020) and most adults will experience this across their lifecourse (Victor, 2021). Consequently it is now acknowledged that loneliness is not just a ‘problem of old age’ or, indeed a ‘natural’ part of the aging process (Victor, 2021). Over the last decade loneliness has been reframed as a major public health problem because of the identification of loneliness as risk factor for premature mortality (Steptoe et al., 2013; Holt-Lunstad et al., 2015), adverse biological parameters (e.g., hypertension), adverse health behaviors (including smoking, excess alcohol consumption and lack of exercise), physical and mental morbidity and increased health service use (Leigh-Hunt et al., 2017).

Dementia is one specific health condition where we can identify two loneliness focused research strands. First there are studies focused upon establishing the prevalence of loneliness in this population. Victor et al. (2020) report that, in a large (*n* = 1445) cohort of adults with mild to moderate dementia, the prevalence of loneliness was 35% (30%, reported being moderately lonely and a further 5% were severely lonely). This approximates to the prevalence in the general population for those aged 65+. The second area of activity focuses upon establishing if loneliness is a risk factor for dementia. Bickel and Cooper (1994) investigated this relationship in a sample of 314 adults aged 65+ in Mannheim followed up for an average of 7.8 years. They reported a non-statistically significant age adjusted relative risk of dementia for those reporting feelings of loneliness of 0.6. (i.e., dementia was 40% lower in lonely versus non-lonely participants).

Since this original paper the number of papers investigating this relationship have increased markedly such that there four systematic reviews published. Boss et al. (2015) reviewed 10 studies, five of which were cross-sectional, and concluded that loneliness is associated with reduced cognitive function but did not report a meta-analysis. Kuiper et al. (2015) included three of their four longitudinal studies in their meta-analysis which reported a statistically significant relationship between loneliness and incident dementia {RR: 1.58 [95% CI: (1.19–2.09)]}. Penninkilampi et al. (2018) reviewed four longitudinal studies and their meta-analysis the relationship between loneliness and dementia risk was not significant [RR: 1.38 (95%CI: 0.98–1.94)]. Lara et al. (2019) concluded that loneliness conferred an increased risk of dementia [RR: 1.26 (95%CI: 1.14–1.4)] based on the eight longitudinal studies included in their meta-analysis. The reviews did not test the relationship between loneliness and dementia using a public health lens as defined by a critical engagement with how the exposure (loneliness) and outcome (dementia) are defined and measured. The aim of our study was to undertake a public health informed analysis of the relationship between loneliness and dementia. This approach emphasizes the relationship between exposure (loneliness) and outcome (dementia) paying particular to how these are defined, conceptualized, and measured. The rationale for this being that existing reviews have not rigorously evaluated these key dimensions to ensure that we are using concepts and terms consistently across studies. We suggest that unless we pay attention to these aspects of studies the evidence for loneliness as a risk factor for dementia and other health outcomes will remain contested.

## Methods

This was not a classical systematic review. Rather we offer a public health analysis as an exemplar of the questions to be asked when evaluating evidence reporting the health consequences of loneliness. We limited our analysis to longitudinal or cohort studies as cross-section studies can establish association but cannot determine if loneliness is a cause or consequence of dementia. We first identified potential papers from the reference lists of the four existing reviews supplemented by a search of six databases: Cochrane Database of Systematic reviews, PubMed, Scopus, CINAHL PsychInfo, Web of Science, and Google Scholar. Our search terms are framed around three key concepts: our exposure of interest-loneliness (loneliness OR lonely OR lonesome or social loneliness or emotional loneliness) our outcome variable (Dementia or Alzheimer’s or cognitive decline or cognitive deficit or cognitive deterioration or cognitive change or cognitive performance OR cognitive status OR cognitive function) and our study type (longitudinal study, cohort or follow-up). These terms were informed by the key words included in previously published reviews.

### Selection of Papers

Papers were included in our analysis if they met six criteria. As dementia is an age-related condition we included studies that related in full or part to people aged 50+ living in the community. Second studies must include a measure of dementia/cognitive decline/cognitive function as the outcome. Thirdly the study must report details of the exposure (loneliness) measure used. Fourthly the studies had to be longitudinal/cohort design of at least 12 months duration in order to have sufficient power to detect new (incident) cases of dementia. Fifthly studies had to exclude those with dementia at baseline from the reporting of the results. Finally studies had to report quantitative data on our outcome, dementia, for lonely vs. non-lonely older adults. No specific start date for studies was set and we included papers up to October 2020. No limitations on study location were set.

Papers were excluded if (a) they did not report loneliness but rather related topics such as isolation or social networks; (b) did not report on dementia but rather related concepts such as mild cognitive impairment; (c) were not longitudinal in design or had a follow period of less than 12 months; (d) were based upon clinical populations, populations of service users, or focused of those aged < 50; (e) did not measure outcome at follow-up or exposure at baseline; (f) did not exclude from the analysis those with dementia at baseline or study did not report quantitative data on dementia comparing lonely and non-lonely older adults.

### Papers Included

We identified 26 papers from existing reviews and database search. All papers were read in full and 13 were excluded because:-(a) no reported measure of loneliness (*n* = 5); duplicate data (*n* = 3); data not reported longitudinally (*n* = 3); did not report new (incident) cases of dementia (*n* = 6). Eleven papers were included in the review.

### Data Extraction

Where studies meet the inclusion criteria, key data addressing four parameters were collected using a standardized extraction form. We extracted methodological details of the cohorts studied including study location, sampling strategy, response rates, attrition, length of follow-up, missing data. We then recorded details of loneliness/dementia risk factors in five categories: (a) demographic (age, sex, marital status, household size); (b) social (isolation, social participation, social networks, (c) physical health (chronic conditions, disability, self-rated health), (d) mental health (depression, cognition) and (e) health behavior (physical activity, smoking, alcohol consumption). Thirdly we extracted details of how our exposure (loneliness) and outcome measures (dementia) were defined and measured. Finally, we extracted details of key results including;– (a) prevalence data for loneliness at baseline; (b) incident dementia and (c) the relationship between loneliness and dementia as relative risk, odds or hazard ratios either crude or adjusted.

## Results

We report our results in five sections: (a) methodological overview of studies identified; (b) the inclusion of co-variates; (c) measurement of our exposure variable; (d) measurement of outcome variable and (e) the evaluation of the relationship between loneliness and dementia.

### Methodological Overview of Included Studies

Our 11 papers were drawn from seven different countries (United States, China, United Kingdom, Singapore, Spain, Sweden, and Netherlands) (Table 1). We included four national Longitudinal Studies of Ageing; The Health and Retirement Survey (HRS) (United States), English Longitudinal Study of Ageing (ELSA), Chinese Longitudinal Health Longevity Survey (CLHLS) are Singapore Longitudinal Study of Aging and six district or city-based cohorts from Amsterdam, Anhui (China), Betula (Sweden), Cambridge (United Kingdom), Shanghai, and Zaragoza (Spain). All 10 of these studies addressed a range of health and wellbeing topics in contrast to the study by Wilson et al. (2007) was unique in specifically focusing upon dementia (Alzheimer’s Disease only). This study recruited participants from churches, low income housing and social service agencies in the Chicago area of the United States. All other studies were based on random community samples derived from primary care registrations/census lists or other population-based sampling strategies.

**TABLE 1:** Longitudinal studies reporting the relationship between loneliness and dementia included in the analysis.

**References**	**Location and population**	**Sample-total number and free of dementia at baseline**	**Follow up**	**Demographic factors-age, gender, marital status, and living alone of follow up samples**	**Co-variates measured**
He et al., 2000	Shanghai, China Population aged 55+	6634 sampled and 5271 (79.5%) responded 96% (5055 people) completed MMSE and 4896 dementia free	1203 followed up at 10 year (25%)	Baseline characteristics not reported	Demographic-age sex
Wilson et al., 2007	Chicago, United States	1023 recruited: 857 (84%) free of dementia	857 followed up annually for 4 years. 791 (92%) completed at least 1 follow-up	Baseline characteristics: Mean age = 80.1 (*SD* = ± 7.1) 76% female;	Demographic, social, physical, and mental health, health behaviors
Lobo et al., 2008	Zaragoza, Spain Population aged 55+	Random sample from census lists. 4803 participated (80% of invited) 4061 free of dementia	3244 followed up at 2 years (90%)	Baseline data for overall sample (*n* = 4061) 58% female Mean age 73.5 (*SD* = ± 9.8)	Demographic-age, sex, education
Chen et al., 2011	Anhui, China-Population aged 60+	Random community sample-*n* = 3336 (response rate 95%); 1637 (took dementia test)	1526 (46% of sample) free of dementia followed up for up to 7.5 years 1307 participated	Baseline characteristic of 1307 at follow-up 44% female 41% aged 65–69; 7% live alone; 18% widowed/divorced	Demographic-age sex
Holwerda et al., 2014	Amsterdam Netherlands Population aged 65–84	Random sample from 30 GPs practices. 4051/5666 (72%) participated at baseline;	Longitudinal 3 year follow up-2173 free of dementia (54% of baseline) participated	Baseline characteristics of dementia free sample 63% female, 51% aged 65–74; 46% live alone; 51% not married	Demographic, social, physical, and mental health, health behaviors
Rafnsson et al., 2017	English Longitudinal Study of Ageing Population aged 50+	Wave 2 of the English Longitudinal Study of Ageing Wave 2, consisted of 8,780 and 6677 formed the analytic sample	Outcomes were assessed in Waves 3 (2006), 4 (2008), 5 (2010), and 6 (2012) for 6,677 participants excluding deaths, missing data and those with cognitive impairment	56% female; Mean age of 66.0 ± 9.4 (SD) (52 to > 90 years); 32% not married;	Demographic, social, physical and mental health
Rawtaer et al., 2017	Singapore-Population aged 55 +	Singapore Longitudinal Study of Ageing 2808 total sample at baseline	2181 dementia free at baseline followed up for up to 8 years-1601 participated (73%)	Baseline characteristics of dementia free sample followed up 7% lived alone 75% were married 65% female Mean age 64.9 (*SD* = + /− 6.8)	Demographic, social, physical and mental health, health behaviors
Sutin et al., 2018	United States- Health and Retirement Survey Population aged 50+	Participants were selected into the analytic sample if they completed the loneliness measure in 2006 or 2008, did not have dementia at baseline, and had at least one follow-up cognitive assessment through the 2016 assessment.	12,030/13,020 participants at baseline included in analysis- followed up for 10 years	Characteristics at baseline Mean age = 67.30 (*SD* = + /− 10.45), 60% female	Demographic, social, physical and mental health, health behaviors
Zhou et al., 2018	China-Chinese Longitudinal Health Longevity Survey	Study started in 1998 with data collection at 5 years intervals up to 2011/12 16954 interviewed in 2008	7867 dementia free and follow up data in 2011/12	Baseline characteristics of follow up sample 45% aged 85 Mean age 83 (range 65–111) 55% female 42% not married 17% living alone	Demographic, social, physical and mental health, health behaviors
Sundström et al., 2020	Betula, Umea, Sweden-Population aged 60+	Longitudinal population cohort started in 1988 with participants selected at random from population registers.	2066 participants baseline and analysis based on Wave 3/4. 1905 (92%) participated and analysis on 1477 free of dementia	Baseline characteristics of follow up sample Mean age = 71.5 (*SD* = /− 9.3) 55% female 38% not married	Demographic, physical and mental health, health behaviors
Wang et al., 2019	Cambridge, United Kingdom aged 75+ cohort	2166 (95%) recruited from primary care in 1985. 10 waves of data collection. Study based on waves 3–10	713 wave 3 participants −102 at wave 10	Characteristics at wave 3 Mean age 86 (*SD* = + /− 4.0) 71% female	Demographic-age, sex, education

We used three key indicators to test the methodological robustness of the studies: response rates, sample attrition and analytical decisions. For the district/city cohorts response rates at baseline ranged from 72% (Holwerda-Amsterdam) to 95% (Chen and Wang, Anhui, China and Cambridge, United Kingdom). Assessing response rates for the nationally based studies, with multiple follow up waves, such as HRS, ELSA, and CLHLS is challenging as these can be calculated for a range of different time points and are not always reported. In longitudinal studies attrition rates, loss of participants from death, inability to follow-up because of relocation, care home admissions, or refusal to participate, reduces both sample size and potentially statistical power and generalizability. The Shanghai cohort reported by He et al. (2000) consisted of 4896 participants free of dementia at baseline but at the 10 years follow only 1203 (25%) of the initial sample were included. The Anhui cohort reported by Chen et al. (2011) offers an illustration of how analytical decisions reduce sample sizes and limit generalizability. Of the initial cohort of 3336 only those with minimal education were included in the dementia testing (1637-49%) of whom 1526 were dementia free-46% of the baseline sample. The analysis is based on 1307 followed up for 7.5 years (mean 3.9): 39% of the initial sample. Studies can also experience decreases in analytical sample sizes because of missing data. Rafnsson et al. (2017) uses wave 2 of ELSA consisting of 8780 participants. However, 1100 of these were excluded because of missing data on the loneliness questions which were included in the self-completion questionnaire rather than the main interview.

Reporting of key demographic characteristics of analytic samples can help assess representativeness, although it was not feasible to evaluate each study individually. Samples in some studies may not be representative of the general population of older adults. For example the study by Wilson et al. (2007) has 70%+ females and high mean age of 80+ which may, or may not, be representative of the communities from which the study volunteers were drawn. Additionally, in evaluating these elements, there are variations across studies in the reporting of demographic parameters. Important factors such as age were reported as mean values [range 86 (Wang et al., 2019) to 66 (Rafnsson et al., 2017)] or percentage in specific age groups (51% aged 65–74 Holwerda et al., 2014) using categories which varied across studies [e.g., % 65–69 by Chen et al. (2011), % aged 85 by Zhou et al. (2018)].

### Measurement of Co-variates

Co-variates are characteristics of populations that may be independently linked with either the outcome or the exposure (or both). The co-variates included were categorized into five groups-demographic (age, sex, marital status, household composition, race, ethnicity, education, employment), health behaviors (physical activity, diet, alcohol consumption, smoking, obesity), social health (isolation, social networks, social participation/engagement, e.g., attending church, groups, activities), physical health (cardiovascular diseases, diabetes, chronic medical conditions, pain, disability) and mental health and wellbeing (depression, cognitive function, quality of life). Studies show considerable variation in the number and range of co-variates included (Table 1 and Supplementary Table S1 for full details). Chen et al. (2011) and Wang et al. (2019) only include socio-demographic factors whilst Wilson, Holwerda, Sutin and Zhou included all groups of co-variates (but not necessarily the same concepts measured consistently). Two key co-variates consistently linked with loneliness are depression, included in five studies and marital status included in four studies. As with demographic factors studies vary in how co-variates are reported.

### Measuring Exposure to Loneliness

Loneliness, our exposure variable, was measured as either a single item question or a scale (see Table 2). Seven studies used single item questions, directly asking participants to rate their loneliness but there was variation in question wording and response categories. Three studies (Sundstrom, Chen, Lobo) used questions that generated a Yes/No response derived from the Geriatric Mental Status schedule (GMS). Holwerda asked if participants felt lonely or very lonely while Zhou et al. (2018) asked if they were never, rarely, sometimes, often or always lonely. Wang et al. (2019) classified participants as not at all lonely, slightly lonely, lonely, and very lonely. Rawtaer et al. (2017) asked participants ‘do you feel at the present moment that you are not at all lonely, fairly lonely or very lonely?’. All single item scores were dichotomized into lonely and not lonely.

**TABLE 2:** Measures of loneliness and dementia included in selected studies.

**References**	**Measure of loneliness**	**Loneliness**	**Measure of dementia**	**Dementia incidence -per 1000 person years at risk (*N*)**
He et al., 2000	Not stated	Loneliness at 10 year follow up: 14% for those without dementia and 22% of those with Alzheimer’s Disease	Clinical interview and MMSE	264 cases of dementia in 10 years follow up-rate not calculated
Wilson et al., 2007	Modified 11 item De Jong Gierveld scale. Five items measuring social loneliness removed; six items measuring emotional loneliness reduced to 5 by combining 2 questions. The items were:- I experience a general sense of emptiness (original wording) I miss having people around (me included in original), I feel like I don’t have enough friends (new question) I often feel abandoned (rejected in original) I miss having a really good (close in original) friend Responses averaged to give a score of 1–5	Total sample mean ± SD, 2.3 ± 0.6; range 1.0–4.6. No AD = 2.2 (0.6) (*n* = 716) AD = 2.5 (0.6) (*n* = 76)	Alzheimer’s Disease only. A composite score based on 19 tests including measures of episodic memory (seven tests), semantic memory (three tests), working memory (three tests), perceptual speed (four tests), and visuospatial ability (two tests)	24 per 1000 (*N* = 76-71 AD, 5 possible AD)
Lobo et al., 2008	Loneliness based on answer to single item question from Geriatric Mental Status question. Responses were loneliness absent (0); loneliness present but mild or not frequent (1) and symptom frequent and/or severe (2). Responses mild or severe combined to create a dichotomous score	Not reported	Cases of dementia identified in the interview (MMSE/GMS) operationalized to match DSM-IV-TR confirmed by clinician	Eighty-two cases of dementia, 47 cases of dementia Alzheimer’s type in 3 years follow. Rates not reported
Chen et al., 2011	Geriatric Mental Status question–feeling lonely with yes/no response categories	Total population = 5% No dementia = 5% (60/1227) Dementia = 9% (7/80)	GMS AGECAT- a computerized diagnostic system, AGECAT (Automated Geriatric Examination for Computer Assisted Taxonomy), designed for use with the Geriatric Mental Status Schedule (GMS). Individuals assigned to diagnosis based on ‘levels of confidence’ ranging from 1 to 5. Participants with score 3+ classed as dementia diagnosis	15.1 per 1000 (*N* = 80)
Holwerda et al., 2014	Do you feel lonely or do you feel very lonely?	Total population = 20% (433/2173) Developed dementia = 37% (58/158) vs. no dementia = 19% (375/2015)	As per Chen et al., 2011 – GMS AGECAT score of 3+	24.2 per 1000 (*N* = 158)
Rafnsson et al., 2017	UCLA scale as per Sutin	Mean UCLA scale (range 1–3) With dementia (220) = 1.54 vs. 1.27 without dementia	Either positive response to question asking about physician diagnosis of dementia or Alzheimer’s disease in past 2 years OR a score of 3.5 + on IQCODE questionnaire if an informant interview	220 (3.3%) reported they were diagnosed with dementia (*n* = 172) or informant rating (*N* = 48) Rate 5.2 per 1000 (*n* = 220)
Rawtaer et al., 2017	Do you feel at the present moment you are not at all lonely, fairly lonely or very lonely (dichotomized in analysis)	17.8% lonely	Dementia defined by modified MMSE using a cut off 27 or below, Clinical Dementia Rating Scale with cases confirmed by clinical review differentiating dementia and mild cognitive impairment (MCI).	163 participants defined as dementia/MCI over up to 8 years follow up-30 per 1000 29 per 1000 for the not lonely group (*N* = 129) and 35 for the lonely (*N* = 34).
Sutin et al., 2018	UCLA 3 item scale: How much of the time do you feel…” and rated each item (“You lack companionship?”, “left out?”, and “isolated from others?”) on a scale from 1 (*often*) to 3 (*hardly ever or never)*	Mean UCLA score = 1.47 (*SD* = + /− 0.54) Mean: no dementia 1.46 (*SD* = + /− 0.53) vs. Incident dementia = 1.59 (*SD* = + /− 0.57)	Score based on 3 items: immediate and delayed recall of 10 words; serial 7 subtraction and backward counting score. Score range 0–27. Dichotomized as either dementia ≤ 6) or not dementia ≥ 7) using validated cut point	1,104 participants (9%) developed dementia Rate 12.4 per 1000
Zhou et al., 2018	Do you feel lonely never, seldom, sometimes, often, always?	Always 2% Often 6% Sometimes 22% Seldom 30% Never 40%	Diagnosis of dementia-are you suffering from dementia and have you been diagnosed with dementia by a physician.	131 developed dementia and 262 experienced this before death at 3-year follow -up-rates not reported
Sundström et al., 2020	Do you often feel lonely yes/no response	Prevalence-total sample = 12% No dementia = 11% Incident dementia = 18% AD = 20% and VD = 16%	Clinical diagnosis, medical records, test results lead by senior geriatric psychiatrist	11.1 per 1000 (*N* = 428)
Wang et al., 2019	Do you feel lonely not at all lonely, slightly lonely, lonely, very lonely? (analysis combines lonely and very lonely)	25% lonely at baseline (W3)-presented by wave but after wave 5 samples very small (*N* = 65 at wave 6 decreasing to 1 at wave)	MMSE score range 0–30 in four categories: normal (26–30), mild cognitive impairment (22–25), moderate cognitive impairment (18–21) and severe (0–17).	

Three studies used a loneliness scale which typically does not directly ask about loneliness. Both Rafnsson et al. (2017) and Sutin et al. (2018) used the three item UCLA loneliness scale with consistent item wording, response options and reporting format (mean score calculated across three questions). This reflects the close links between ELSA and HRS in terms of questions asked. Wilson et al. (2007) reports using the 11 item de Jong Gierveld (DJG) scale with modification. The scale as designed and validated has five items measuring social loneliness and six emotional loneliness rated on a 3-point scale. In the adaptation the social loneliness items were removed and the response format changed to 5-point scale. The six emotional loneliness items were reduced to five by combining two questions to form a new question; three questions had wording changes and one was as per the published scale. These changes are substantial and preclude comparison with other studies that have used the scale as developed.

These variations across the 11 studies making comparisons of loneliness prevalence at baseline across studies or population norms challenging. Zhou et al. (2018) reports that 7.6% of the sample were often or always lonely while Rawtaer et al. (2017) reported that 18% of their sample were fairly or very lonely at the present moment. For self-report measures using a yes/no response format- the overall prevalence of loneliness varied from 5% (Chen-China) to 12% (Sundstrom-Sweden). Holwerda et al. (2014) reports for The Netherlands a baseline prevalence of 20% for those reporting that they were lonely/very lonely.

### Measuring Outcome-Dementia

There are four approaches to the conceptualization and measurement of our outcome variable, dementia: clinical diagnosis, diagnostic screening tools, cognitive function tests and self-reported diagnosis (Table 2). Clinical diagnosis and tests, linked to MMSE and/or GMSAGECAT scores, are used in five studies (Lobo, Sundstrom, Wilson, He, and Rawtaer) Two studies used a dementia screening tool either the Mini Mental State examination (Wang) or the Geriatric Mental Status (Chen and Holwerda) without clinical confirmation of diagnosis. Sutin, used four tests of cognitive function (immediate and delayed word recall, serial 7 subtraction and reverse counting). Rafnsson et al. (2017) used self-reported diagnosis by a GP in previous 2 years and Zhou et al. (2018) asked participants if they were suffering from dementia and if this had been diagnosed by a doctor.

Dementia incidence is reported as cases per 1000 person years at risk. This ranges from 5 per 1000 (Rafnsson et al., 2017), which used self-reported doctor diagnosis, to 24 per 1000 (Holwerda and Wilson). Incidence rates vary between studies using similar methods of assessing dementia. The two studies using the GMS AGECAT method report rates per 1000 of 15.1 (Chen) and 24.2 (Holwerda). Similar variation is demonstrated by studies using clinical diagnosis ranging from 11.1 (Sundstrom) to 24 per 1000 (Wilson-Alzheimer’s disease only).

#### What Is the Relationship Between Loneliness and Dementia?

The relationship between loneliness and dementia is presented in four different ways in our 11 papers (Table 3). Five studies report odds ratios (OR), two use relative risk (RR) and three use hazard ratios (HR) and one a multistate model. Relative risk compares the incidence or risk of an event (dementia) among those with/without a specific exposure (loneliness). Odds ratios compare the presence/absence of an exposure (loneliness) to a specific outcome (dementia). Hazard ratios introduce a temporal element and consider how outcomes, in our case dementia, for two groups (lonely vs. not lonely) change relative to each other over time. At the most basic for all three measures a rate of 1 indicates no difference between groups. The 95% confidence intervals show the range or precision of the estimates and where these cross 1 the result is not significant. For all three indicators reports can be reported for crude and adjusted rates the latter considering the influence of co-variates.

**TABLE 3:** Relationship between loneliness and dementia.

**References**	**Analysis Crude and adjusted ratios for dementia**	**Evaluation of relationship**
He et al., 2000	Crude Relative Risk 1.63 (95% CI: 0.93–2.86)	No significant relationship
Wilson et al., 2007	Relative Risk = 1.45 (95% CI: 1.01–2.09)-adjusted for age, sex, education, social network, social activity Relative Risk = 1.41 (95% C: 0.99–2.01) after controlling for cognitive activity, age, sex, education	Once cognitive activity is controlled for there is no relationship. Study has a robust outcome measure for Alzheimer’s disease but based on a volunteer sample and used a highly modified loneliness measure
Lobo et al., 2008	Odds Ratio for dementia = 3.25 (95% CI: 0.92–11.5) adjusted for age, sex, education, 23 non-cognitive symptoms	Result reported in reported in Lara et al. (2019) -relationship not significant
Chen et al., 2011	Odds Ratio = 1.69 (95% CI: 0.79–3.89) adjusted for age/sex-NS	No significant relationship
Holwerda et al., 2014	Crude Odds Ratio 2.56 (95% CI: 1.82–3.61) Adjusted Odds Ratio 1.64 (95% CI: 1.05–2.56)	Statistically significant result after adjustment for key factors:- age, sex, education, marital status, living alone, depression, cognitive function, social support, disability cardiovascular, and other medical conditions. Robust measure of outcome.
Rafnsson et al., 2017	Adjusted Odds ratio 1.36 (95% CI: 1.03 – 1.80)	Statistically significant result adjusted for age, education, marital status, physical health, social isolation, close relationships, depression but outcome is self-report of doctor diagnosis.
Rawtaer et al., 2017	Crude Hazard Ratio 1.26 (95% CI: 0.86–1.84) Adjusted ratio: 1.02 (95% CI: 0.66–1.57)	No significant relationship
Sutin et al., 2018	Hazard Ratio = 1.40 (95% CI: 1.26–1.56) (adjusted for age, gender, race, ethnicity, and education) Hazard Ratio = 1.19 (95% CI:1.05–1.33) (additional adjustment for hypertension, diabetes, smoking, BMI, isolation, depression).	A significant relationship but the outcome is cognitive function rather than dementia.
Zhou et al., 2018	Odds Ratio 1.15 (95% CI: 1.05–1.33) Age, sex, area, social isolation, health behaviors, marital status, living alone, cognitive function, disability, chronic diseases	The result is significant, but the outcome is based upon self-reported diagnosis/deaths from dementia.
Sundström et al., 2020	Reported three outcomes: all dementia and Alzheimer’s (AD) and Vascular (VaD) separately Age sex adjusted models using Hazard Ratios All-cause dementia (HR = 1.46, 95% CI 1.14–1.89) AD (HR = 1.69, 95% CI 1.20–2.37) VaD (HR = 1.34, 95% CI 0.87–2.08) Additional adjustment for education, marital status, smoking, alcohol use, previous cardiovascular diseases/disorders, and depressive symptoms all-causes dementia (HR = 1.38, 95% CI 1.04–1.84) AD (HR = 1.83, 95% CI 1.25–2.67) VaD (Hazard Ratio 1.08 95%CI: 0.66–1.77)	Outcome significant for all cause and Alzheimer’s disease but uses a dichotomous exposure measure.

Six studies did not report a statistically significant relationship between loneliness and dementia in either crude or adjusted analyses (He, Wilson, Lobo, Chen, Rawtaer, and Wang). Five studies reported a significant relationship between loneliness and dementia (Holwerda, Rafnsson, Sutin, Zhou, and Sundstrom) with the excess ‘risk’ of dementia amongst those who were lonely compared with the non-lonely ranging from 15% to 64%. It is, however, difficult to make direct comparisons across these studies as there are four different measures of both outcome and exposure. Sutin and Rafnsson use the same measure of loneliness but different dementia outcomes and co-variates. Zhou and Rafnsson use a similar dementia outcome, self-report of doctor diagnosis, but different exposure and co-variates. The heterogeneity demonstrated by our 11 studies precluded a robust meta-analysis.

## Discussion

There is a considerable interest in the relationship between loneliness and a range of health outcomes, one of which is dementia. Determining causality using observational data in the absence of experimental evidence is challenging. However, conducting randomized controlled trials is in the field of loneliness research is both impractical and unethical. Consequently we used the approach of Howick et al. (2009, 2019) based on the criteria established by Hill (1965) to examine the relationship between loneliness and dementia using a public health lens. We augmented existing reviews by focusing on how exposure (loneliness) and outcome (dementia) were defined and measured alongside the inclusion in the analysis of appropriate co-variates as well as study design. We included only longitudinal studies of samples free of dementia to ensure that the exposure (loneliness) predated the outcome (dementia: a prerequisite for determining causation rather than association.

Studies varied considerably in terms of key study design quality parameters of response and representativeness, sample size and attrition. The four national longitudinal studies of aging are the most robust in terms of overall study quality being the largest and most representative but, like other studies included, experience attrition, issues of missing data and decrease in power resultant from analytical decisions. Reporting of key factors demonstrated variability across studies. For example, demographic composition of samples was reported in three ways: percentage of older adults (aged 60 or 65+), percentage in a specific age group (e.g., 60–69) or as mean age. This makes both establishing representativeness and cross-study comparisons problematic, especially when dealing with age-related outcomes.

We can extend our public health approach to examining the relationship between loneliness and dementia further by testing the quality of the evidence using the framework developed by. This generated a set of 8 guidelines to test if relationships, in his case between smoking and lung cancer, were likely to be causal. We focus on the aspects of the Hill (1965) criteria of strength and coherence of evidence with respect to measurement and inclusion of co-variates, the measures of outcome and exposure and study results.

Our studies are heterogeneous in terms of covariates, loneliness (exposure) and dementia(outcome) measurement. We identified five domains of co-variates: socio-demographic, social health, health behaviors, physical health and mental health. Four studies, Wilson, Holwerda, Sutin, and Rawtaer, included all domains while three included on socio-demographic factors (He, Lobo, and Wang) and five studies did not include depression: one of the key factors linked to loneliness.

While all studies measure loneliness and not related concepts such as isolation they do so in different ways making cross study comparison challenging. However, there are broader issues than just simple question used. Loneliness is complex-there are different types of loneliness and current measures favor social rather than emotional or existential loneliness (Mansfield et al., 2019). Measures largely focus on intensity of loneliness but there are other components of the experience such as frequency and duration which remain largely unexplored in terms of their influence on health outcomes. The studies included in our analysis report a one off loneliness measure using it to predict an outcome of dementia up to 20 years in the future. Is that plausible? Longitudinal studies that measure loneliness at different time points and can differentiate between different loneliness trajectories may be a more powerful way examining these relationships (Zhong et al., 2016). The presence of a dose-response relationship is key for public health in terms of relationship plausibility, but the measures used in the papers which are largely dichotomous preclude this type of inference.

We see similar heterogeneity in how dementia is measured ranging from clinical examinations, the use of screening tools, measures of cognitive function and self-reported diagnoses. This limits our confidence in the results when we are not consistently using the same outcome measure. Of our 10 studies that presented results in a similar way 5 show a significant relationship but there is no consistency across these in terms of the measurement of exposure, outcome and inclusion of co-variates. The revised Bradford Hill criteria suggest that strength of evidence is demonstrated by an effect size of 1.5: this was demonstrated by only 1 study (Holwerda). In combination these issues suggest we should be cautious in making claims for causation.

Our review has highlighted several challenges in evaluating the relationship between loneliness and dementia in terms of heterogeneity in measures of exposures and outcomes which may apply more widely. It is, however, important to note that the large scale longitudinal studies of aging which can investigate the loneliness-health relationships include multiple domains and are not designed with this focus as their sole purpose. One response would be that such studies adopt a consistent set of measures of loneliness and key health outcomes. In the United Kingdom as part of the national loneliness strategy we have adopted two loneliness measures-a self report question and the short UCLA scale (DCMS, 2018). In the absence of such a consensus, then attempting to generate ‘a common currency’ of loneliness measurement so that results can be compared across studies would be a valuable methodological contribution to the field. Similarly a suite of common health measures that can be adopted in portmanteau surveys focused on, for example, cognitive function could strengthen our knowledge base and enable us to determine if loneliness is a cause or consequence of dementia. The studies included in this analysis report findings about dementia. However we see that there are ranges of different ways this is measured from clinical diagnosis to cognitive function. The latter is not a measure of dementia but indicates a potential trajectory toward dementia.

Our research agenda extends beyond simple issues of measurement consistency. We need to recognize the complexity of loneliness. To date our evidence for the effect of loneliness on health outcomes is based around measures of social loneliness and the frequency with which it is experienced. Extending our understanding to include emotional and existential loneliness and the inclusion of duration and frequency of loneliness may give a fuller picture of the impact of loneliness. A further fruitful research area is looking at lifecourse loneliness exposure and how this may link to health in later life. Most current research looking at health outcomes uses a baseline loneliness exposure measurement. It is worth exploring if trajectories of loneliness-differentiating chronic from transient may-influence health outcomes rather than a simple one off measurement.

One reason why loneliness has attracted such attention is that it is seen as being modifiable and therefore a route to changing health outcomes. Current evidence suggests that interventions to reduce loneliness or the onset of loneliness are of limited effectiveness (Victor et al., 2018) and there is a need for further research in this area (National Academies of Sciences, Engineering, and Medicine, 2020). Parallel to this we need to develop our understanding of the underlying pathways or mechanisms, physiological, biological, psychological or social by which loneliness affects health outcomes. This, for example, could be directly via inflammation or indirectly via mediated links such as poor sleep (Kidambi and Lee, 2020; Kim et al., 2020). Understanding the mechanisms by which loneliness effect health is a key component of the research agenda for the next decade.

## Author Contributions

CV designed, undertook, analyzed, and wrote up the study.

## Conflict of Interest

The authors declare that the research was conducted in the absence of any commercial or financial relationships that could be construed as a potential conflict of interest.

## Supplementary Material

Supplementary Table 1 Inclusion of co-variates in studies.
