# Supplementary material for: Is Loneliness a Cause or Consequence of Dementia? A Public Health Analysis of the Literature
Source: Front Psychol. 2021 Feb 18;11:612771. doi: 10.3389/fpsyg.2020.612771 (PMC7929990; doi:10.3389/fpsyg.2020.612771)
Supplement: Supplementary Table 1 — Inclusion of co-variates in studies. [file Table_1.docx]

Table S1: Inclusion of co-variates in studies

| *Reference* | Socio-demographic | Social  health | Health behaviours | Physical health/  disability | Mental health |
| --- | --- | --- | --- | --- | --- |
| He et al. 2000 | Age, sex |  |  |  |  |
| Wilson et al. 2007 | Age, sex, education, race, ethnicity, income | Social networks,  Social participation | Physical activity | Vascular burden (CVD) and vascular risk factors, Disability | Depression-CESD-10 item (reports sores for 9 items-loneliness question removed)  Cognitive function at baseline |
| Lobo et al. 2008 | Age, sex, education |  |  |  |  |
| Chen et al. 2011 | Age and sex |  |  |  |  |
| Holwerda et al. 2014 | Age, sex, marital status, living alone, education | Social support | Alcohol, smoking | Medical conditions-CAMDEX questions, IADL/ADL | Depression using AGECAT, cognitive function |
| Rafnsson et al. 2017 | Age, sex, education, marital status | Social isolation, close friendships |  | Hypertension,diabetes, stroke, cancer, cvd, mobility | Depression -CESD 8 item with loneliness question removed  Cognition index |
| Rawtaer et al. 2017 | Age, sex, marital status, ethnicity, living alone, education | Quality of life, social activities, productive activities | Smoking, alcohol,  obesity | Hypertension,  diabetes, | Depression-geriatric depression scale |
| Sutin et al. 2018 | Age, sex, education, race, ethnicity | Social isolation | Physical activity, smoking, BMI | Hypertension, diabetes-self report of doctor diagnosis | Depressive symptoms-CESD-8 with loneliness question removed |
| Zhou et al. 2018 | Age, education level, employment status, living alone, marital status, education, rural | Social support | Physical activity, smoking, alcohol | Cardiovascular disease, diabetes, ADL and IADL | Cognitive function |
| Sundstrom et al. 2020 | Age, sex, education, marital status | , | Smoking, alcohol | Cardiovascular disorders, diabetes | Depression-CESD 20 item-loneliness item removed |
| Wang et al. 2019 | Age, sex, education |  |  |  |  |
